# Electron microscopic recording of myosin head power stroke in hydrated myosin filaments

**DOI:** 10.1038/srep15700

**Published:** 2015-10-26

**Authors:** Haruo Sugi, Shigeru Chaen, Tsuyoshi Akimoto, Hiroki Minoda, Takuya Miyakawa, Yumiko Miyauchi, Masaru Tanokura, Seiryo Sugiura

**Affiliations:** 1 Department of Physiology, School of Medicine, Teikyo University, Itabashi-ku, Tokyo 173-8605, Japan; 2 Department of Integrated Sciences in Physics and Biology, College of Humanities and Sciences, Nihon University, Setagaya-ku, Tokyo 156-8550, Japan; 3 Department of Applied Physics, Tokyo University of Agriculture and Technology, Koganei-shi, Tokyo 184-8588, Japan; 4 Graduate School of Agricultural and Life Sciences, University of Tokyo, Bunkyo-ku, Tokyo 113-0032, Japan; 5 Graduate School of Frontier Sciences, University of Tokyo, Kashiwa-shi, Chiba 277-8563, Japan

## Abstract

Muscle contraction results from cyclic attachment and detachment between myosin heads and actin filaments, coupled with ATP hydrolysis. Despite extensive studies, however, the amplitude of myosin head power stroke still remains to be a mystery. Using the gas environmental chamber, we have succeeded in recording the power stroke of position-marked myosin heads in hydrated mixture of actin and myosin filaments in a nearly isometric condition, in which myosin heads do not produce gross myofilament sliding, but only stretch adjacent elastic structures. On application of ATP, individual myosin heads move by ~3.3 nm at the distal region, and by ~2.5 nm at the proximal region of myosin head catalytic domain. After exhaustion of applied ATP, individual myosin heads return towards their initial position. At low ionic strength, the amplitude of myosin head power stroke increases to >4 nm at both distal and proximal regions of myosin heads catalytic domain, being consistent with the report that the force generated by individual myosin heads in muscle fibers is enhanced at low ionic strength. The advantages of the present study over other *in vitro* motility assay systems, using myosin heads detached from myosin filaments, are discussed.

More than 60 years have passed since the monumental discovery that muscle contraction results from relative sliding between actin and myosin filaments, coupled with ATP hydrolysis^1^,^2^. It is now widely accepted that the myofilament sliding is caused by cyclic attachment-detachment between the myosin heads extending from myosin filaments and the sites on actin filaments^3^. Concerning performance of the myosin heads in producing the myofilament sliding, it is generally believed that a myosin head first attaches to actin, change their structure, i.e. power stroke, to produce myofilament sliding, and then detaches from actin^4^. Biochemical studies on the actomyosin ATPase reaction steps in solution^5^ indicate that the myosin head (M), in the form of M · ADP · Pi, first attaches to actin (A), and performs a power stroke, associated with release of Pi and ADP, so that M forms rigor (or rigor-like) linkage with A at the end of power stroke. Upon binding with a new ATP, M detaches from A, performs a recovery stroke, associated with reaction, M · ATP → M · ADP · Pi, and again attaches to actin.

A most effective approach to the structural changes of a myosin head may be to determine the unitary distance of myofilament sliding caused by a myosin head power stroke. In the contraction model of A.F. Huxley and Simmons^6^, each myosin head is assumed to have elastic element, while the myofilaments are rigid; when a muscle fiber contracts isometrically, its isometric tension results from the tension in stretched elastic element, which resides in individual myosin heads. The amount of extension of myosin head elastic element is therefore equal to the amplitude of myosin head power stroke (or step size) in the isometric condition, and can be estimated from the amount of quick decrease in fiber length to drop the isometric force to zero (~1% of fiber length or ~10 nm/half sarcomere)^6^. The above amplitude of myosin head power stroke is, however, proved to be an overestimate, since it has been shown that myofilaments are not rigid, but have finite elasticity^7^,^8^,^9^. Experiments to estimate the amplitude of myosin head power stroke have been made by activating skinned muscle fibers in rigor state with the laser flash photolysis of caged ATP^10^,^11^. Although the values obtained are within the framework of the Huxley-Simmons contraction model^6^ , these results are obscured by uncertainties concerning the proportion of myosin heads involved in fiber shortening. Similar attempts have also been made with *in vitro* motility assay systems, especially with highly sophisticated optical trap experiments^12^ and X-ray diffraction studies on contracting muscle^13^,^14^,^15^. However, no definite conclusion has been reached concerning the amplitude of myosin head power stroke by these studies, because of random orientation of myosin heads interacting with actin. Recently, Kaya and Higuchi estimated the myosin head step size by analyzing stepwise movement of actin filament past myosin heads in myosin filament^16^. However, their analysis is indirect, since it stands on a step-finding algorithm.

Apart from these approaches, a most straightforward way to determine the power stroke amplitude in individual myosin heads is to use the gas environmental chamber (or hydration chamber) attached to an electron microscope. As early as 1997, Sugi *et al.*^17^ succeeded in recording the ATP-induced movement of individual myosin heads, position-marked with gold particles via antibody to myosin head, in hydrated myosin-paramyosin core complex in the absence of actin filaments. Using the same method, they further succeeded in recording the ATP-induced recovery stroke in individual myosin heads in hydrated myosin filaments prepared from rabbit skeletal muscle, and showed that the amplitude myosin head recovery stroke, associated with reaction M + ATP → M · ADP · Pi, was ~6 nm^18^. This constituted the first success in directly determining the amplitude of recovery stroke in individual myosin heads, moving freely in hydrated myosin filaments retaining their physiological function. Furthermore, using three different antibodies to position-mark different regions within a myosin head, Minoda *et al.*^19^ demonstrated that the amplitude of recovery stroke was similar between the distal and the proximal regions of myosin head catalytic domain (CAD), in which both actin-binding and ATPase cites are located. These findings indicate that, even in the absence of actin filaments, each myosin head can undergo its structural changes without being guided by actin filaments.

In the present experiments, we attempted to record the ATP-induced power stroke in individual myosin heads in hydrated mixture of actin and myosin filaments. The amplitude of myosin head power stroke, recorded in nearly isometric condition, was found to be ~3.3 nm and ~2.5 nm at the distal and the proximal regions of myosin head CAD, respectively. When, on the other hand, the external KCl concentration was reduced from 125 to 20 mM, the amplitude of myosin head power stroke increased to >4 nm at both the distal and the proximal regions, in agreement with our physiological finding that the force exerted by individual myosin heads in Ca^2+^-activated skinned muscle fibers increases twofold at low ionic strength^20^. As the amplitude of power stroke in individual myosin heads in the isometric condition is expected to increase with increasing force produced by individual myosin heads, the present results may constitute the first experimental success in directly recording myosin head power stroke in the physiological condition.

## Results

### Stability of Myosin Head Position in the Absence of ATP

**Figure 1:**
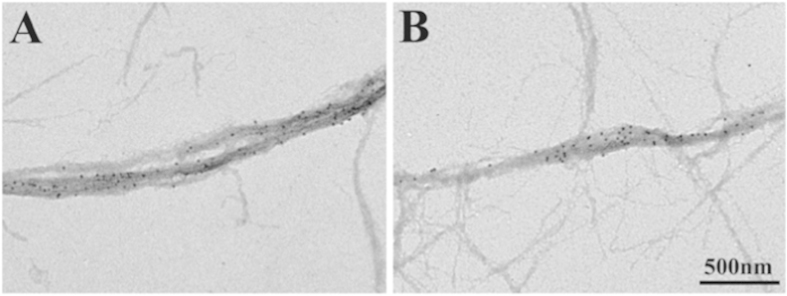
Low magnification conventional electron micrographs showing part of actin and myosin filament mixture. Myosin heads were position-marked with antibody 1 in the left micrograph, and with antibody 2 in the right micrograph. Thick myosin filaments, with gold particles attached to myosin heads, are surrounded by thin actin filaments.

**Figure 2:**
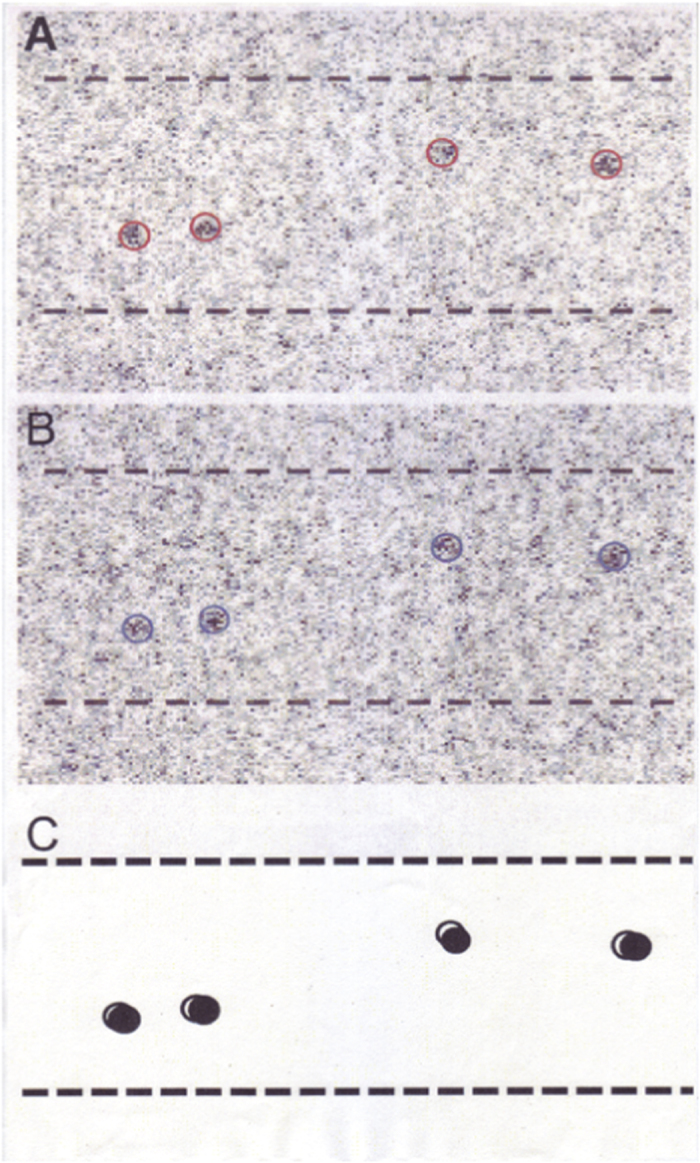
Example of a pair of IP records of the same myosin filament in the filament mixture (A,B). Record A was taken before ATP application, and record (**B**) was taken after ATP application. Circles (diameter, 20 nm; red in (**A**), and blue in (**B**) are drawn around the center of mass positions of individual gold particle image, consisting of a number of dark pixels. (**C**) Diagram showing ATP-induced changes in position of gold particles attached to individual myosin heads with antibody 1. Open and filled circles (diameter, 20 nm) are drawn around the center of mass positions of the same particles, before and after ATP application, respectively. Broken lines indicate approximate contour of myosin filaments.

First, we observed mixture of actin and myosin filaments under a low electron microscopic magnification, after negative staining with 1% uranyl acetate. Though the mixture of actin and myosin filaments showed a tendency to aggregate, we could occasionally observe actin filaments not only running in random directions, but also running in parallel with thick myosin filaments, with a number of gold particles attached to myosin heads via antibodies 1 or 2 (Fig. 1). Since the number of actin filaments was in large excess of the number of myosin filaments, majority of myosin heads in each myosin filament were expected to form rigor linkages with actin in the absence of ATP. During experiments with hydrated filament mixture (magnification, 10,000 X) under unstained conditions, we could observe gold particles attached to myosin heads and approximate contour of myosin filaments, while actin filaments were invisible (see Fig. 2A,B).

To ascertain whether the particle position, i.e. the myosin head position, changes with time or not, we took two imaging plate (IP) records of the same myosin filament at intervals of 5–10 min without ATP application, and compared changes in the center of mass position of the same particles, attached to individual myosin heads, between the two IP records. As has been the case in the previous experiments^17^,^18^,^19^, the distance (D) between the two center of mass positions of the same particle, i.e. the change in position of each position-marked myosin head, was very small. Among the 220 particles examined on 5 different pairs of IP records, 86 particles showed D values <0.5 nm which were regarded to be insignificant, and the rest 134 particles showed D values between 0.5 and 2 nm. The average D value was 0.6 ± 0.06 nm (mean ± SD, n = 220). These results indicate that the filament mixtures are firmly fixed in position on the carbon sealing film, and that, despite thermal fluctuations, the mean position of individual myosin head CAD, time averaged over 0.1 s, does no change appreciably with time in the absence of ATP. The stability of myosin head mean position provides a favorable condition to determine the amplitude of ATP-induced myosin head movement unambiguously. The range of variation of myosin head position changes with time observed in the present study was much smaller than that observed in the absence of actin filaments^17^,^18^,^19^, supporting the idea that, in the presence of actin filaments, majority of myosin heads form tight actin-myosin linkages. Since individual myosin heads stay at or around their equilibrium position^18^, it follows naturally that, in the filament mixture, myosin heads bound to actin filaments bear little or no tension.

### Recording of ATP-Induced Myosin Head Power Stroke in the Mixture of Actin and Myosin Filaments

We examined the ATP-induced myosin head movement in the filament mixture by comparing a pair of IP records of the same filament, taken before and after ATP application. Taking the diffusion time of ATP from the electrode to the filaments into consideration^17^,^18^, the second record was taken at 40–60 s after the onset of current pulse to the electrode, while the first record was taken 2–3 min before the onset of current pulse to the electrode. Figure 2A,B is an example showing a pair of IP records of the same myosin filament with myosin heads, position-marked with gold particles. Records A and B were taken before and after application of ATP, respectively. In both records A and B, a circle of 20 nm diameter (red in A and blue in B) are drawn around the center of mass position of each gold particle image, consisting of a number of dark pixels^18^,^19^. In Fig. 2C, open and filled circles (diameter, 20 nm) show myosin head positions before and after ATP application, respectively. It can be seen that myosin heads exhibit small but distinct position changes in response to applied ATP.

In the present study, the ATP concentration around individual myosin heads was estimated to be 5–10 *μ*M^17^,^18^, so that, after completion of an ATP-induced movement, the myosin head have to wait for next ATP for a considerable period of time (probably >0.5 s). The above intermittent ATP supply to myosin heads has made it possible to record position changes of individual myosin heads when they repeat their power and recovery strokes, despite limited time resolution of our recording system(exposure time to record filament image, 0.1 s). This experimental condition resembles that of optical trap experiments, in which a single myosin head is made to interact with an actin filament at *μ* molar ATP concentrations; unitary force spikes (duration, 0.2–2 s) are recorded at intervals of >0.5 s^12^,^21^.

### Amplitude of ATP-induced Myosin Head Power Stroke

**Figure 3:**
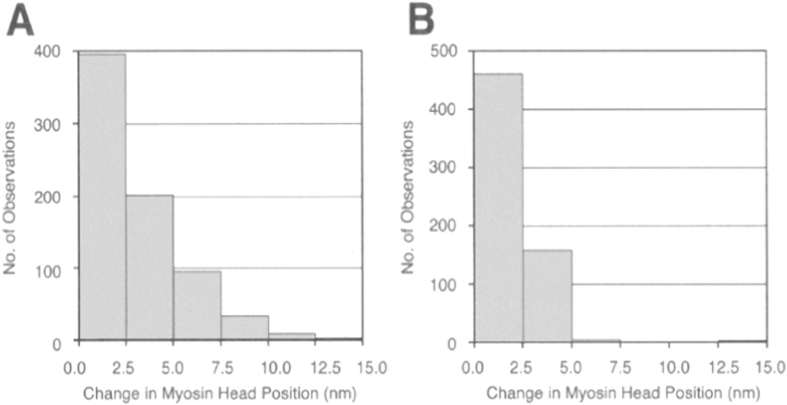
Histograms showing the amplitude distribution of ATP-induced myosin head power stroke at standard external KCl concentration (120 mM). Individual myosin heads were position-marked with antibody 1 at the distal region of myosin head CAD in (**A**), and with antibody 2 at the proximal region of myosin head CAD in (**B**).

By using antibodies 1 or 2 to attach gold particles to individual myosin heads, it was possible to measure the amplitude of power stroke at the distal or the proximal region of myosin head catalytic domain. Figure 3 is a histogram showing the amplitude distribution of ATP-induced myosin head power stroke at the distal region (Fig. 3A) and at the proximal region (Fig. 3B) of myosin head catalytic domain. Considering the small concentration of iontophoretically delivered ATP (5–10 *μ*M), only a very small proportion of myosin heads can be activated by ATP, while the rest of myosin heads remain bound to actin filaments. Consequently, myosin heads power stroke can not produce gross filament sliding, but can only stretch adjacent elastic structures (including elasticity of myosin head^6^ and myofilaments^7^,^8^,^9^). As a matter of fact, the amplitude of ATP-induced myosin head movement never exceeded 15 nm in the present study, indicating the absence of gross myofilament sliding. In this sense, ATP-induced myosin head movement may be analogous to myosin head movement in muscle fibers with two ends fixed, i.e. nominally (or nearly) isometric condition.

The histograms A and B in Fig. 3 are constructed from 732 measurements on 5 different pairs of IP records obtained from 5 different filaments mixtures, and from 613 measurements on 4 different pairs of IP records obtained from 4 different filament mixtures, respectively. The average amplitude of ATP-induced myosin head power stroke was 3.3 ± 0.2 nm (mean ± SD, n = 732) at the distal region, and 2.5 ± 0.1 nm (mean ± SD, n = 613) at the proximal region of myosin head catalytic domain. These results indicate that, in the present experimental condition, where gross myofilament sliding does not take place, the amplitude of power stroke is larger at the distal region than at the proximal region of myosin head catalytic domain (*t* test, p < 0.01). These values are much smaller than those obtained in conditions, where myosin heads move freely in the absence of actin filament^18^,^19^, constituting the proof that, myosin head movement recorded in the present study actually corresponds to myosin head power stroke, though actin filaments are invisible in the IP records.

Recently, we have found that the isometric force generated by individual myosin heads in skinned muscle fibers is enhanced ~twofold by reducing the ionic strength of contracting solution by reducing external KCl concentration from 125 to ≤20 mM^20^. In this work, the fibers were made to contract with two ends fixed, so that myosin heads perform power stroke to stretch adjacent elastic structures, and this condition is similar to that in the present experiments. It is therefore expected that, at low ionic strength, individual ATP-activated myosin heads may stretch adjacent elastic structures more markedly when they perform power stroke.

**Figure 4:**
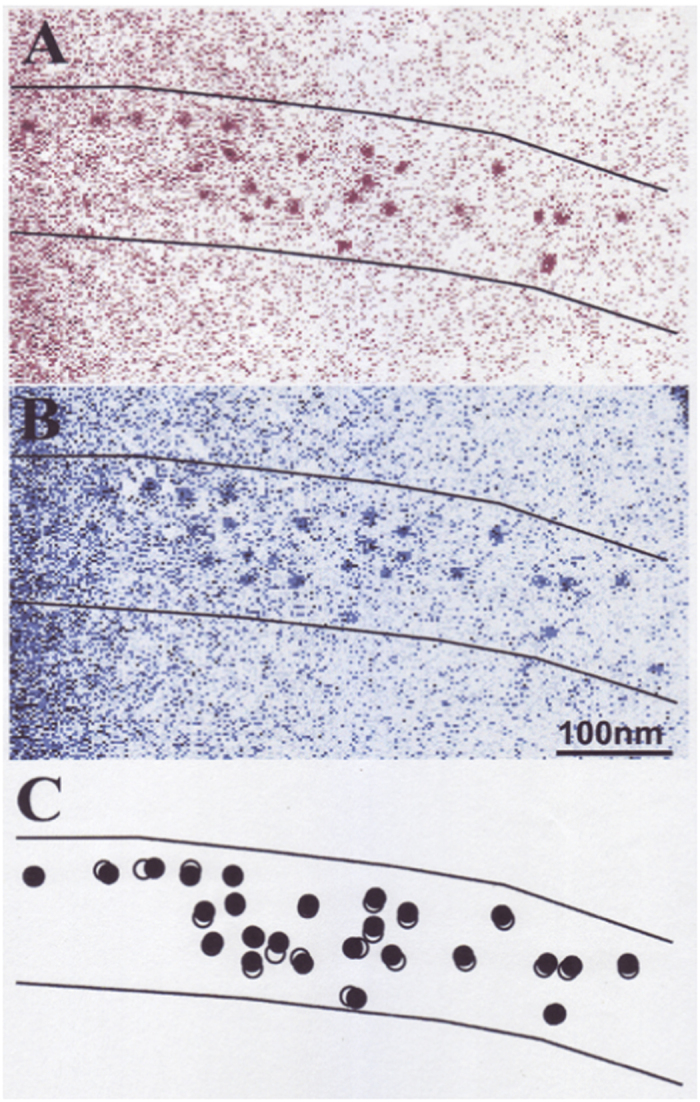
Example of a pair of IP records of the same myosin filament in the filament mixture (A,B). Record A (colored red) was taken before ATP application, and record B (colored blue) was taken after ATP application. Recordings were made at low external KCl concentration (20 mM). (**C**) Diagram showing ATP-induced changes in position of gold particles attached to individual myosin heads with antibody 2. Open and filled circles (diameter, 20 nm) are drawn around center of mass positions of the same particles before and after ATP application, respectively. Solid lines show approximate contour of myosin filament.

**Figure 5:**
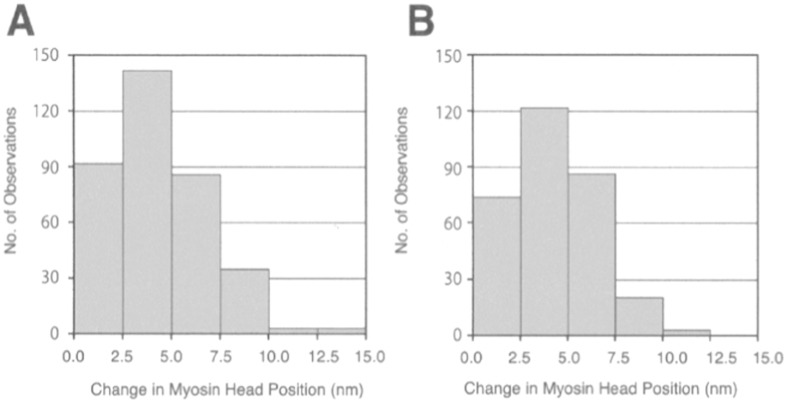
Histograms showing the amplitude distribution of ATP-induced myosin head power stroke at external KCl concentration of 20 mM. Individual myosin heads were position-marked with antibody 1 at the distal region of myosin head CAD in (**A**), and with antibody 2 at the proximal region of myosin head CAD in (**B**).

To test the validity of this expectation, we performed experiments, in which the KCl concentration of experimental solution surrounding the filament mixture was lowered from 120 to 20 mM (corresponding to a reduction of ionic strength *μ* from 170 to 50 mM). Examples of IP records taken before and after ATP application at low ionic strength are shown in Fig. 4. In this particular case, the IP record of gold particles attached to individual myosin heads, taken before ATP application is colored red (A), and that taken after ATP application is colored blue (B), while the bottom diagram (C) shows ATP-induced change in position of each gold particle, i.e. the ATP-induced power stroke in individual myosin heads. The amplitude of ATP-induced myosin head power stroke was found to increase markedly by the reduction of ionic strength. In contrast with the histograms shown in Fig. 3, the histograms of myosin head power stroke at low ionic strength exhibited distinct peaks at 2.5–5 nm around both distal and proximal regions of myosin head catalytic domain (Fig. 5A,B). The average amplitude of myosin head power stroke at low ionic strength was 4.4 ± 0.1 nm (mean ± SD, n = 361) and 4.3 ± 0.2 nm (mean ± SD, n = 305) at the distal and at the proximal regions of myosin head catalytic domain, respectively, indicating that the amplitude of myosin head power stroke does not differ significantly between the distal and the proximal regions of myosin head catalytic domain at low ionic strength.

In the present study, we measured the amplitude of power stroke as externally recorded changes in position of gold particles, i.e. individual myosin heads. In the present experimental condition, ATP-activated myosin heads can move by stretching adjacent elastic structures. The amplitude of myosin head power stroke, recorded in the present study, may therefore be expected to increase with increasing force generated by individual myosin heads. The marked increase in the amplitude of power stroke at low ionic strength (Fig. 5) is entirely consistent with our previous report that, at low ionic strength, the force generated by individual myosin heads increases ~twofold in contracting muscle fibers^20^.

### Reversibility of the ATP-Induced Myosin Head Power Stroke

**Figure 6:**
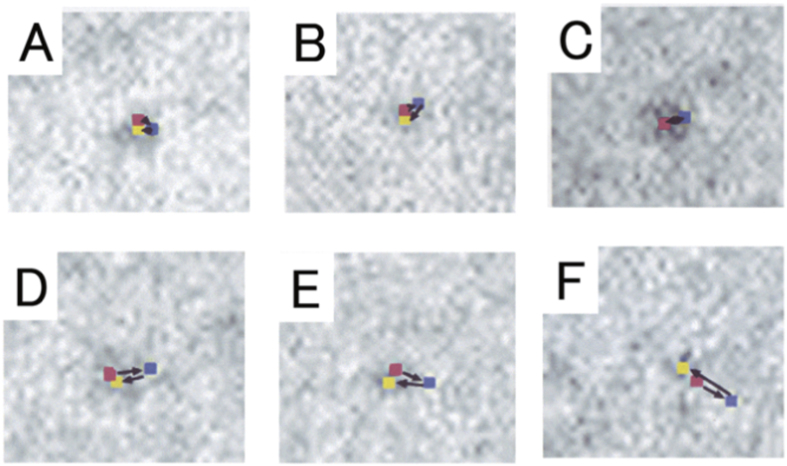
Examples showing sequential changes in position of different pixels (each 2.5 × 2.5 nm) where the center of mass positions of corresponding different particles are located. In each frame, pixel positions were recorded three times, i.e., before ATP application (red), after ATP application (blue), and after complete exhaustion of applied ATP (yellow). Direction of each pixel movement, i.e. direction of myosin head movement, is indicated by arrows. Frames (**A**,**C**,**D**) were obtained from myosin heads position-marked with antibody 1, while frames (**B**,**E**,**F**) were obtained from myosin heads position-marked with antibody 2.

To ascertain whether the ATP-induced myosin head power stroke in the filament mixture is reversible or not, experiments were performed in which the images of the same filament was recorded three times in the following sequence: (i) before the ATP application, (ii) 40–60 s after the onset of current pulse to the ATP-containing microelectrode, i.e. after completion of the ATP-induced myosin head power stroke, and (iii) 5–6 min after ATP application, i.e. after complete exhaustion of applied ATP. Since the experimental solution contained hexokinase and D-glucose serving as scavenger for ATP^17^,^22^,^23^, all the ATP molecules released from the electrode were completely exhausted until the time of recording. Similar results were obtained irrespective of whether individual myosin heads were position-marked by antibody 1 or antibody 2. Figure 6 shows examples of sequential changes in position of 9 different pixels (2.5 × 2.5 nm), where the center of mass potions of the corresponding 9 particles are located. The pixel positions in the first, second and third IP records are colored blue, red and yellow, respectively. Similar results were obtained from myosin heads irrespective of whether they were position-marked with antibody 1 or antibody 2.

When the amplitude of myosin head movement was ≤5 nm, the myosin heads returned almost exactly to their position before ATP application, as indicated by the complete overlap of the pixel positions in the first and third records, so that yellow pixels were completely covered by blue pixels (Fig. 6A–D). With larger amplitudes of ATP-induced myosin head movement, the myosin heads tended to return to the position close to their initial position, as indicated by the small distance between red and yellow pixels (Fig. 6E,F).

These results indicate that the ATP-induced myosin head movement, with amplitudes ≤5 nm, is reversible, Interpretation of the results described so far will be discussed later.

## Discussion

Using the gas environmental chamber, which enables us to study structural changes of wet biomolecules retaining their physiological function electron microscopically, we have succeeded in recording ATP-induced power stroke in individual myosin heads extending from myosin filaments. The advantage of the present EC experiment over *in vitro* motility assay experiments including the optical trap methods is that it can record ATP-induced movement in individual myosin heads extending from hydrated myosin filaments. From the standpoint of muscle physiology, it is essential to study function of myosin molecules in the state similar to that in muscle; in the EC experiment, myosin molecules constitute myosin filaments similar to those in muscle, while *in vitro* motility assay experiments myosin heads are detached from myosin filaments at the LD-S-2 boundary, and it is ambiguous how detached myosin heads are fixed in position. Therefore, the experimental condition in our EC system is much closer to that in muscle compared to that *in vitro* motility assay systems.

**Figure 7:**
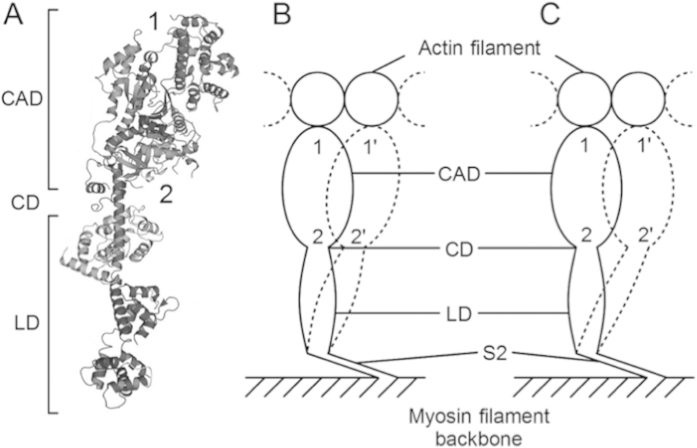
Change in the mode of myosin head power stroke depending on experimental conditions. (**A**) Diagram of myosin head structure consisting of catalytic domain (CAD), converter domain (CD), and lever arm domain (LD). Approximate attachment regions of antibody 1 and antibody 2 are indicated by numbers 1 and 2, respectively. (**B**) The mode of myosin head power stroke in the isometric condition (external KCl, 120 mM). The amplitude of movement is larger at the distal region than at the proximal region of myosin head CAD. (**C**) The mode of myosin head power stroke in the isometric condition (external KCl, 20 mM). The amplitude of movement is similar around both the distal and the proximal regions of myosin head CAD.

We have already successfully performed the EC experiments to record ATP-induced myosin head recovery stroke in the absence of actin filaments^18^,^19^, One may, however, wonder whether the ATP-induced movement of gold particles, attached to myosin heads via antibodies, actually reflects movement of myosin heads per se. We believe that the gold particle movement is similar or close to the myosin head movement for the following reasons. (1) Both the isometric force development and the force-velocity relation in Ca^2+^-activated rabbit psoas muscle fibers remain unchanged in the presence of antibodies 1 and 2, which can diffuse into muscle fibers to effectively bind with their epitopes^24^. (2) The amplitude of gold particle movement in the absence of actin filament does not differ significantly irrespective of whether myosin heads are position-marked by antibody 1 or antibody 2^19^. This result is entirely consistent with the generally accepted lever arm mechanism^25^, in which myosin head catalytic and converter domains move parallel to the long axis of myosin filament. (3) The present experiments have also shown that, in the presence of actin filaments, the amplitude of gold particle movement does not differ significantly irrespective of whether myosin heads are position-marked by antibody 1 or antibody 2 (Figs 5 and 7C), indicating that the lever arm mechanism holds in ATP-induced myosin head power stroke, provided the force generated by individual myosin head are enhanced at low ionic strength^20^. These results indicate that, except for myosin head movement at standard ionic strength (Fig. 3), myosin head movement takes place parallel to the myosin filament long axis, precluding the possibility that myosin heads move radially so that their motion is amplified by antibodies and gold particles extending from them. In Fig. 4, some particles are seen to move normal to myosin filament long axis, but such movement can be accounted for to be due to irregular arrangement of actin-myosin filament mixture.

In our work using the EC, filaments were firmly fixed on the surface of carbon film. One may wonder whether myosin head located at the filament lower surface may not move or move only slightly by being fixed in position. Fortunately, in our previous experiments^17^,^18^,^19^, myosin heads are regarded to move in response to applied ATP irrespective of whether they are located at the upper or lower surface of myosin filaments, since histograms of amplitude distribution of myosin head movement exhibits a distinct peak at >2.5 nm, though bead movements were measured at random; if myosin heads located at the lower surface are immobilized, histograms would also show a peak at <2.5 nm. This indicates that myosin filaments are partially, but not evenly, fixed to carbon film, thus allowing myosin head located their lower surface to move in response to ATP. The same explanation would apply for the present experiments; a proportion of myosin heads as well as actin filaments, located at the lower surface of actin-myosin head mixture would be able to interact in response to applied ATP. Of course, it should be taken into consideration, that myosin head power stroke might be reduced in amplitude at the lower surface of the mixture, implying that we underestimate values of power stroke amplitude in the present study.

We could record myosin head positions at three different states, i.e. (1) before ATP application, (2) during ATP application, and (3) after exhaustion of applied ATP (Fig. 6). When the distance between position 1 and position 2 was small (<5 nm), the distance between position 1 and position 3 was zero (Fig. 6C) or very small (Fig. 6A–C). If the distance between position 1 and position 2 was large (>10 nm), the distance between positions 1 and 3 was large (Fig. 6D,E,F). These results can be taken to indicate that both positions 1 and 3 correspond to myosin head position at pre-power stroke or non-force generating state, while position 2 corresponds to post-power stroke or force-generating state. The large distance between positions 1 and 3 (Fig. 6D,E,F) may be explained as being due to changes in the filament mixture network due to non-uniform distribution of ATP-activated myosin heads. On ATP application, a region with high myosin head density (high force region) pulls another region with low myosin head density (low force region), so that myosin heads in the low force region will be displaced to some extent. The resulting changes in structure of the filament mixture network may remain after exhaustion of applied ATP. If this explanation is correct, the large distance between myosin head positions 1 and 2 > 10 nm may bear no direct relation with the amplitude of myosin head power stroke. Thus, if the nonuniform myosin head density in the filament mixture is taken into consideration, it may be safe to conclude that individual myosin heads take two stable states during repeated attachment-detachment cycle with actin; i.e. pre-power stroke or non-force generating state and post-power stroke or force generating state. This conclusion agrees with the general expectation that, in order to repeat power and recovery stroke in the isometric condition, the amplitude of recovery stroke should be the same as that of power stroke. In this connection, large amplitude recovery stroke (~6 nm) recorded in the absence of actin filaments^18^ might corresponds to that during free loaded muscle shortening, in which the amplitude of myosin head power stroke is largest, probably ~6 nm.

As already mentioned in the Results, the experimental condition in the present study is analogous to that in the optical trap experiments^12^,^21^ in that myosin heads, attached to actin filaments, are activated with ATP at *μ* molar concentrations. Due to a long time until next ATP comes to attach myosin heads, myosin heads held in the optical trap alternately takes force generating and non-force generating states, which show up as intermittent force spikes that can be recorded on a slow time base. Although myosin heads are not firmly trapped in position in the present study, the amplitude of ATP-induced myosin head movement was in many cases <5 nm. Since myosin heads repeat power and recovery strokes many times during the period of IP recording, this result is also consistent with the view that power and recovery strokes are the same in amplitude.

It has been reported that the force generated by individual myosin heads in skinned muscle fibers increases ~twofold at low ionic strength^20^. This report is consistent with the definite increase in the amplitude of ATP-induced myosin head movement at low ionic strength (Figs 2, 3, 4, 5); as the force generated by individual myosin heads in the filament mixture is also expected to increase at low ionic strength, the distance of adjacent elastic structures pulled by them increases to result in increase in the amplitude of myosin head power stroke (Fig. 5).

Figure 7A is a ribbon diagram showing the structure of myosin head (or myosin subfragment 1, S-1), consisting of catalytic domain (CAD), containing actin-binding and ATPase sites, and lever arm domain (LD), connected to myosin filament backbone via subfragment-2 (S-2). The CAD and the LD are connected with converter domain (CD). Mainly based on crystallographic studies on nucleotide-dependent structural changes of S-1, it has been suggested that the CAD is rigid, while the CD serves as flexible joint between the CAD and the LD; myosin head power stroke results from active rotation of the LD around the CD, so that the whole pear-shaped CAD moves parallel to the myosin filament long axis during myosin head power stroke. Such a mode of myosin head power stroke is called the lever arm mechanism^25^.

Contrary to the lever arm mechanism, however, the present results showed that, in the nominally isometric condition at standard ionic strength (120 mM KCl), the amplitude of power stroke was significantly larger at the distal region (~3.3 nm) than at the proximal region of myosin head CAD (~2.5 nm)(Fig. 3). On the other hand, the amplitude of power stroke was not different between the distal and the proximal regions of myosin head CAD in the nearly isometric condition at low ionic strength (20 mM KCl) (Fig. 5), being consistent with the lever arm mechanism. Assuming that all myosin head domains and S-2 are rigid, the two possible modes of myosin movement before (solid line) and after (dotted line) power stroke are shown diagrammatically in Fig. 7B and C. In Fig. 7B, myosin head power stroke is produced mainly by rotation of myosin head around the LD-S-2 junction in such a way that the amplitude of movement is larger at the distal region than at the proximal region of myosin head CAD. In Fig. 7C, on the other hand, myosin head power stroke results from rotation of the CAD around the CD-LD junction (or the CD) as well as rotation of the LD around the LD-S-2 junction, so that the CD moves parallel to the myosin filament axis.

Although the two different modes of myosin head power stroke can also be accounted for by assuming flexibility of myosin head domains, the present findings suggest that the mode of myosin head power stroke changes depending on the conditions; myosin head power stroke takes place by the lever arm mechanism when the force generated by individual myosin heads is enhanced at low ionic strength^20^ (Fig. 7C), but not at standard ionic strength (Fig. 7B). In this connection, it has been reported that Ca^2+^-activated force generation is inhibited by antibodies to myosin S-2 or myosin head LD without changing MgATPase activity^24^,^26^. Much more experimental work is needed to settle this point. In addition, it is of interest to note that, in the optical trap experiments on the amplitude of power stroke of myosin heads in myosin filaments, Kaya and Higuchi^16^ show a diagram of myosin head configuration under high external load, which resembles that shown in Fig. 7B not only in appearance but also in power stroke amplitude (~3 nm), though their analysis is model-dependent.

In summary, we performed electron microscopic recording of ATP-induced myosin head power stroke in hydrated mixture of actin and myosin filaments. Due to limited amount of iontophoretically applied ATP, activated myosin heads pulled adjacent elastic structures without causing gross filament sliding. The amplitude of power stroke in the nearly isometric condition was ~3.3 nm and ~2.5 nm at the distal and the proximal region of myosin head CAD. At low ionic strength, the amplitude of power stroke increased to ~4 nm at both the distal and the proximal regions of myosin head CAD. This work constitutes the first success in visualizing movement of individual myosin heads extending from myosin filaments, while all other studies on myosin head movement have been made using myosin heads detached from myosin filaments.

## Methods

### The gas environmental chamber

The gas environmental chamber (EC) used was identical to that of Sugi *et al.*^17^,^18^ and Minoda *et al.*^19^. The EC is a cylindrical metal compartment (diameter, 2.0 mm; height, 0.8 mm) with upper and lower windows to pass electron beam. Each window is covered with a thin carbon film (thickness, 10–20 nm) supported on a copper grid with 9 apertures. The filament mixture sample was placed on the lower carbon film, and covered with a thin layer of experimental solution (thickness, ~250 nm), which was in equilibrium with circulating water vapor through the EC (pressure, 60–80 torr; temperature, 26–28 ^o^C). In this way, the filament samples were kept in wet, living state for more than 2 hours. The EC contained an ATP-containing glass microelectrode with its tip immersed in the experimental solution. The EC was attached to a 200 kV transmission electron microscope (JEM 2000EX, JEOL).

### Preparation of Actin and Myosin Filament Mixture

White male rabbits weighing 2 to 2.5 kg were killed by injection of sodium pentobarbital (50 mg/kg) into the ear vein, and psoas muscles were dissected from the animals. The animals were treated in accordance with the Guiding Principles for the Care and Use of Animals in the Field of Physiological Sciences, published by the Physiological Society of Japan. The protocol was approved by the Teikyo University Animal Care Committee (protocol 307–050). Actin filaments (F-actin) were prepared from rabbit muscles by the method of Spudich and Watt^27^. Myosin was also prepared from rabbit psoas muscle by the method of Perry^28^, while myosin rod was prepared by chymotryptic digestion of myosin by the method of Margossian and Lowey^29^. Both myosin and myosin rod were frozen and stored in 50% (vol/vol) glycerol at −20 ^o^C until used. Myosin and myosin rod were mixed at a molar ratio of 1:1, and slowly polymerized by dialysis against a solution of low ionic strength to obtain bipolar myosin filaments (length, ~1.5–3 *μ*m; diameter at the center, 30–200 nm). Details of preparation of the synthetic myosin filaments have been described elsewhere^18^. Actin and myosin samples were mixed in such a way that actin filaments were in large excess of myosin filaments.

### Position-marking of individual myosin heads

Colloidal gold particles (diameter, 20 nm; coated with protein A; EY Laboratories) were attached to myosin heads in myosin filaments as position markers, using two different site-directed antibodies (IgG); one (antibody 1) to junctional peptide between 50- and 20-kDa segments of myosin heavy chain^30^, while the other (antibody 2) to reactive lysine residue (lys 83), located at the boundary between catalytic and converter domains of myosin head^31^. Antibodies 1 and 2 were used to position-mark the distal and the proximal regions of myosin head CAD (cf. Fig. 7A), respectively. The antibodies attach to only 1 of the 2 myosin heads probably because of steric hindrance^18^,^19^,^30^. To label myosin heads sparsely, so that each gold particles could be clearly distinguished from neighboring ones, the molar ratio between myosin heads in myosin filament and antibody was chosen to be ~1: 1.4–1.6^18^,^19^. Further details have been described^17^. Finally, mixtures of actin and myosin filaments were prepared by mixing actin filament (5 *μ*M) and myosin filament (12.5 *μ*M) samples at a volume ratio of 2:1.

### Recording of Myosin Head Position in Filament Mixture

To avoid electron beam damage to the filaments, observation and recording were made with a total incident electron dose <10−^4^ C/cm^2^ (or 6.2 × 10^−2^ electrons / square Angstrom), being well below the critical dose to eliminate ATP-induced myofibrillar shortening^32^. For this purpose, the filament mixtures were observed with extremely weak beam intensities <5 × 10^−13^ A/cm^2^ at the detector. The actual beam intensity through the filaments with a magnification of 10,000 × was 5 × 10^−13^ × (10,000)^2^ = 5 × 10^−5^ A/cm^2^. As soon as the filament images, with gold particles serving as myosin head position markers, were brought in focus, electron beam was stopped except for the time of recording. The filament mixture images were recorded with an imaging plate (IP) system (PIX system, JEOL) with a magnification of 10,000 x. The exposure time was 0.1 s with a beam intensity of 1–2 × 10^−12^ A/cm^2^. Because of the limitation of total incident electron dose, recording of the same filaments can be made only ~2–4 times^17^,^18^,^19^.

### Application of ATP

The application of ATP to the filament mixture was made iontophoretically by applying a negative current pulse (intensity, 10 nA; duration, 0.5–1 s) from an electronic stimulator to a glass capillary microelectrode filled with 100 mM ATP through a current clamp circuit^29^,^30^. A positive DC current was constantly applied to the electrode to inhibit spontaneous release of ATP from the electrode. The total amount of ATP released from the electrode was estimated to be ≤10^−14^ mol. Assuming the volume of experimental solution covering the filament mixtures of ~10^−6^ mL, the ATP concentration around the filament mixture was 5–10  *μ*M. The time required for the released ATP to reach the filament mixture by diffusion was estimated to be <30 s, by recording the ATP-induced shortening of myofibrils mounted in the EC under a light microscope. To record the filament mixture images after complete exhaustion of applied ATP, hexokinase (50 units/mL) and D-glucose (2 mM) were added to the experimental solution as scavengers for ATP^18^,^22^,^23^.

### Data Analysis

The filament mixture images recorded on the IP were analyzed with a personal computer. Under a magnification of 10,000 ×, the pixel size on the IP was 2.5 × 2.5 nm, and the average number of electrons reaching each pixel during the exposure time was ~10. Reflecting this electron statistics, each gold particles image consisted of 8–20 dark pixels. After contrast enhancement and binarization procedures, particle with approximately round configurations were selected for analysis. The center of mass position for each selected particle was determined as the coordinates (two significant figures; accuracy ~0.5 nm) within a single pixel where the center of mass position was located. These coordinates, representing the position of the particle, i.e., the position of the myosin head, were compared between 2 different IP records of the same filament mixture. The absolute coordinates common to the 2 IP records were obtained from the position of natural markers (bright spots on the carbon film). The distance (D) between the two center of mass positions (with the coordinates X_1,_ Y_1_ and X_2_ Y_2_, respectively) was calculated as D = √{(X_1_–X_2_)^2^ + (Y_1_–Y_2_)^2^}^17^,^18^,^19^.

## Additional Information

**How to cite this article**: Sugi, H. *et al.* Electron microscopic recording of myosin head power stroke in hydrated myosin filaments. *Sci. Rep.* **5**, 15700; doi: 10.1038/srep15700 (2015).
